# Circular RNAs and glioblastoma multiforme: focus on molecular mechanisms

**DOI:** 10.1186/s12964-021-00809-9

**Published:** 2022-01-28

**Authors:** Raziyeh Salami, Marziyeh Salami, Alireza Mafi, Omid Vakili, Zatollah Asemi

**Affiliations:** 1 Department of Clinical Biochemistry, School of Medicine, Hamedan University of Medical Sciences, Hamedan, Iran; 2 Department of Clinical Biochemistry, School of Medicine, Shahid Sadoughi University of Medical Sciences, Yazd, Iran; 3 Department of Clinical Biochemistry, School of Pharmacy and Pharmaceutical Sciences, Isfahan University of Medical Sciences, Isfahan, Iran; 4 Research Center for Biochemistry and Nutrition in Metabolic Diseases, Institute for Basic Sciences, Kashan University of Medical Sciences, Kashan, Iran

**Keywords:** Glioblastoma multiforme, Circular RNA, Signaling pathways, Diagnosis, Treatment

## Abstract

Glioblastoma multiforme (GBM), as a deadly and almost incurable brain cancer, is the most invasive form of CNS tumors that affects both children and adult population. It accounts for approximately half of all primary brain tumors. Despite the remarkable advances in neurosurgery, radiotherapy, and chemotherapeutic approaches, cell heterogeneity and numerous genetic alterations in cell cycle control, cell growth, apoptosis, and cell invasion, result in an undesirable resistance to therapeutic strategies; thereby, the median survival duration for GBM patients is unfortunately still less than two years. Identifying new therapeutics and employing the combination therapies may be considered as wonderful strategies against the GBM. In this regard, circular RNAs (circRNAs), as tumor inhibiting and/or stimulating RNA molecules, can regulate the cancer-developing processes, including cell proliferation, cell apoptosis, invasion, and chemoresistance. Hereupon, these molecules have been introduced as potentially effective therapeutic targets to defeat GBM. The current study aims to investigate the fundamental molecular and cellular mechanisms in association with circRNAs involved in GBM pathogenesis. Among multiple mechanisms, the PI3K/Akt/mTOR, Wnt/β-catenin, and MAPK signaling, angiogenic processes, and metastatic pathways will be thoroughly discussed to provide a comprehensive understanding of the role of circRNAs in pathophysiology of GBM.

**Video Abstract**

**Supplementary Information:**

The online version contains supplementary material available at 10.1186/s12964-021-00809-9.

## Background

Glioblastoma multiforme (GBM) is still known as a deadly brain cancer and accounts for approximately half of all primary brain tumors [1]. Currently, the resection surgeries, followed by radiotherapy and temozolomide (TMZ)-based chemotherapy are the major approaches to cure the GBM. However, high rates of cell heterogeneity and multiple genetic alterations in cell cycle control, cell growth, apoptosis, and cell invasion have complicated these therapeutic proceedings, and subsequently have led to therapeutic resistance. Therefore, GBM has remained an incurable malignancy with an average survival duration of about 12–15 months [2, 3]. Due to the mentioned issue, it is critical to identify novel therapeutic targets and combination therapies to become more powerful against the GBM and its undesirable effects. Biomarkers actually help us to monitor patients at different stages of treatment, select an appropriate strategy, and improve the efficiency of GBM therapy [4, 5]. In this regard, several studies have suggested the non-coding ribonucleic acids (ncRNAs) as amazing diagnostic, prognostic, and also therapeutic targets in cancer therapy [6–8].

NcRNAs are defined as a large group of RNA molecules with no potential of being translated, and can be classified into two subclasses based on their size; (I) short ncRNAs that are smaller than 200 nucleotides in size (e.g. microRNA (miRNA) with a length of about 20 to 23 nucleotides), and (II) long ncRNAs (lncRNAs) with a length of more than 200 nucleotides [9]. Circular RNAs (circRNAs) are located in the second subfamily. These circular RNA molecules have covalently closed-loop structures without 5ʹ-to-3ʹ polarity [10]. They also possess some amazing characteristics, which have made them protected against the exonucleolytic digestion [11]. CircRNAs are large in number; they are stable structures, and are widely distributed in multiple tissues, cell types, and biological fluids, making them to be easily detectable [12, 13].

Several studies have shown that metabolic and genetic disorders there are in patients with diabetes and cancer [14–16]. In addition, circRNAs can exert modulatory effects on cancer-related processes, such as tumorigenesis, tumor progression, and apoptosis [17, 18]. Additionally, researchers have reported a strong correlation between the expression of circRNAs and various cancers, suggesting that circRNAs may act as tumor-inhibiting and/or promoting molecules [19]. In this context, numerous investigations have assessed the function of different circRNAs in GBM cell proliferation, migration, invasion, and apoptosis, and thus these molecules have been introduced as promising therapeutic targets to combat GBM [20–22]. Hence, a thorough understanding of circRNAs and their roles on GBM-related signaling and molecular mechanisms may result in identification of more powerful therapeutic strategies. Therefore, the current review aims to investigate the fundamental mechanisms and signaling pathways affected by circRNAs during the progression of GBM.

## Pathophysiology of GBM

Among various malignant gliomas, GBM, which accounts for about 60–70% of all gliomas, is the most invasive form of the CNS tumors that can affect both children and adult population [23]. GBM is most commonly located inside the cerebral hemispheres, especially in the supratentorial region, and only a small percentage of tumors occur in the brain stem, cerebellum, and spinal cord [24]. According to the World Health Organization (WHO) classification, GBM is described based on the molecular characteristics, histological parameters, and clinical manifestations [25]. GBMs are histologically defined as diffuse astrocytomas with the highest degrees of malignancy; so called the astrocytoma grade IV [26]. Moreover, these tumors are characterized by prominent features, including the increased cell proliferation, angiogenesis, necrosis, significant resistance to apoptosis, and extensive genomic changes [23, 27].

Another categorization of GBMs is based on its clinical characteristics. In this classification, there are two subclasses; primary GBMs which are usually arisen de novo, during 3–6 months, and secondary GBMs which develop gradually from the low grade astrocytomas, over a 10 to 15-year period [28]. Primary GBM is the most prevalent form (~ 95%) and is often occurred in elderly patients, while the secondary GBM usually affects younger individuals [29]. Primary and secondary GBMs have similar histological features but represent some genetic and epigenetic variations; the overexpression of epidermal growth factor receptor (EGFR), mouse double minute 2 (MDM2) gene mutation and amplification, p16 deletion, and phosphate and tensin homologue (PTEN) mutation, are the hallmark alterations of primary GBMs, whereas the overexpression of the platelet-derived growth factor A (PDGFA) and the PDGF receptor alpha (PDGFR), and the mutations of the isocitrate dehydrogenase1/2 (IDH1/2) and the tumor protein p53 (TP53) are mostly frequent in secondary GBMs [30–32]. These mutations and genetic alterations will eventually lead to an uncontrolled cell proliferation, an increased cell survival, and the escape of tumor cells from cell cycle checkpoints, aging processes, and apoptotic pathways [33].

The Cancer Genome Atlas (TCGA) has categorized GBMs into four molecular subclasses, including the classical, mesenchymal, proneural, and neural, based on the genomic and proteomic analyses [34]. The identification of these molecular alterations will provide prospects to improve the current therapeutic strategies and develop a new model to manage this life-threatening malignancy. Classical GBM is defined by EGFR mutation or amplification, as well as PTEN and p16INK4A deletion. The mesenchymal subtype is characterized by the mutations and/or loss of the TP53, neurofibromin-1 (NF1), and cyclin-dependent kinase inhibitor 2A (CDKN2A). On the other hand, the mutations of PDGF, along with the IDH1/IDH2, p53, phosphoinositide-3-kinase catalytic subunit alpha (PI3KCA), and phosphoinositide-3-kinase regulatory subunit 1 (PI3KR1) mutations are the main clarifying features of proneural subtype. Finally, the neural GBM is described without any particular genetic signature [31, 34].

## The landscape of circRNAs

### The biogenesis and regulation of circRNAs

CircRNAs are a substantial group of ncRNAs with covalently closed-loop structures without 5ʹ-to-3ʹ polarity that are generated from pre-mRNA during the back-splicing or exon skipping, the processes that are different from the canonical splicing [35, 36]. CircRNAs can be derived from exons, introns, untranslational regions (UTRs), antisense RNAs, and intergenic regions [37]. These molecules are classified into three major subtypes based on their inner elements; exonic circRNAs (EcircRNAs), which contain exons only; exon–intron circRNAs (EIciRNAs), which are derived from both of introns and exons; and circular intronic RNAs (ciRNAs) which are generated from introns [38]. EcircRNAs account for the major portion of circRNAs, and are mostly found inside the cytoplasm. On the contrary, ciRNAs and EIciRNAs are mainly present within the nucleus [39].

In the case of circRNA biogenesis, there are two speculative models that are broadly accepted; first, lariat-driven circularization, in which pre-mRNA is exposed by partial splicing due to its closeness to the exon-donor site and different exon acceptor sites on the same locations, resulting in the skipping of one or more exons [40]. This process accelerates the formation of a lariat intermediate that contains a large amount of exons and introns. The introns are then separated, followed by the attachment of an upstream exon with a downstream exon, resulting in the formation of exon-derived circRNAs [41]. However, under the certain conditions, intronic sequences are retained, leading to the generation of EIciRNA [42]. Furthermore, this mechanism also promotes the ciRNA biogenesis using a GU-rich 7-nucleotide sequence near the 5' splice site and a C-rich 11-nucleotide motif close to the 3' branch point [40]. The second mechanism is Intron-pairing driven circularization, in which a circular composition is generated by complementary pairing on both sides of the introns, consisting of repetitive sequences like the *Alu*, leading to the formation of different types of circRNAs, including the EcircRNAs and EIciRNAs [43].

Like for linear RNAs, the biogenesis of circRNAs is controlled by RNA binding proteins (RBPs), including the muscle blind (MBL), quaking (QKI), double-stranded RNA editing enzyme- adenosine deaminase acting-on RNA (ADAR), and the nuclear helicase DHX9 [44]. MBL and QKI bind to pre-RNA to bring the splicing sites into a close proximity, inducing the generation of circRNAs [45, 46]. ADAR can convert adenine to inosine, declines the RNA complementarity, and unwinds the stem, preventing from circRNA formation [47, 48]. Similarly, DHX9 also suppresses the biogenesis of circRNAs; since this RBP comprises of an RNA-binding domain and a helicase RNA domain, is able to open the RNA pairs to inhibit the circRNA expression. Therefore, the concomitant depletion of ADRA and DHX9 can up-regulate the expression of circRNAs [47].

### Biological functions of circRNAs

The biological functions of circRNAs are closely associated with their specific structures and molecular features. The well-understood functions of these RNA molecules are classified as follows:

#### CircRNAs as miRNA sponges

MicroRNAs (miRNAs), as the other substantial ncRNA molecules, are able to induce the mRNA degradation or translation suppression via attaching to the target sites through the mRNA’s 3′- UTR [49]. CircRNAs have interestingly been reported to contain particular miRNA response elements (MREs), which provide the structural basis for their function as molecular sponges. They also prevent the interaction between miRNAs and mRNAs, which indirectly affect the downstream target genes and the subsequent production of proteins [50, 51]. A group of circRNAs greatly tend to bind to miRNAs and are referred to as "super sponges." One of the well-studied examples of super sponges is the ciRS-7, which has more than 70 binding sites for miR-7. Thereby, it works as a miRNA sponge and affects the miR-7’s target mRNA transcripts [50, 52]. Moreover, there are many other circRNAs that can act as miRNA sponges, such as the circHIPK3 [53], circPVT1 [54], cir-ZNF609 [55], circ-MMP9 [56], etc. [57].

#### Interaction with proteins

CircRNAs, which contain particular binding sites for RBPs, can specifically bind to particular proteins and act as protein sponges to change the activity of those proteins [58]. For instance, the circ-Foxo3 has a strong affinity for some of the aging- and stress-associated transcription factors, such as the inhibitors of differentiation 1 (ID-1), E2F transcription factor 1 (E2F1), hypoxia-inducible factor-1α (HIF-1α), and focal adhesion kinase (FAK). The circ-Foxo3 can reduce the nuclear translocation of ID-1, E2F1, and HIF-1α, as well as the mitochondrial translocation of FAK during the cardiac stress, which induces the heart aging process [59]. CircRNAs also play critical roles as protein scaffolds for two or more proteins through their binding site, just like the circ-Foxo3 that is able to bind to the cyclin-dependent kinase inhibitor 1 (CDK1 or p21) and the CDK2 in order to facilitate the p21-induced inhibition of CDK2 and suppressing the cell cycle progression through the G1 phase [60].

#### The modulation of parental gene expression

CircRNAs located inside the nucleus, including the CiRNAs and EIciRNAs, are able to operate the parental gene expression at both transcriptional and post-transcriptional levels [61]. EIciRNAs, such as the circ-EIF3J and circ-PAIP2 were found to deal with the U1 small nuclear ribonucleic proteins (snRNPs) and RNA polymerase II to induce the expression of the parental gene [42]. Additionally, the ci-Ankyrin Repeat Domain 52 (ci-ANKRD52) can interact with RNA polymerase II (Pol II), and subsequently increases the transcription of corresponding genes [40]. Interestingly, when EIciRNAs and/or ciRNAs are downregulated, the transcription efficiency of their host genes becomes decreased. These findings may help researchers to understand why EIciRNAs and ciRNAs are found mainly inside the nucleus. Further evaluations have shown that circRNAs can also be involved in the modulation of post-transcriptional processes, such as the selective splicing. A study, performed by Ashwal-Fluss et al., indicated that circMbl might compete with MBL pre-mRNA for selective splicing. Indeed, since MBL protein contains specific binding sites for circMbl, MBL can deal with circMbl in order to increase its biogenesis. Thus, circMbl down-regulates the canonical splicing and declines the generation of the functional mRNAs [45].

#### Potential translation capability

**Fig. 1:**
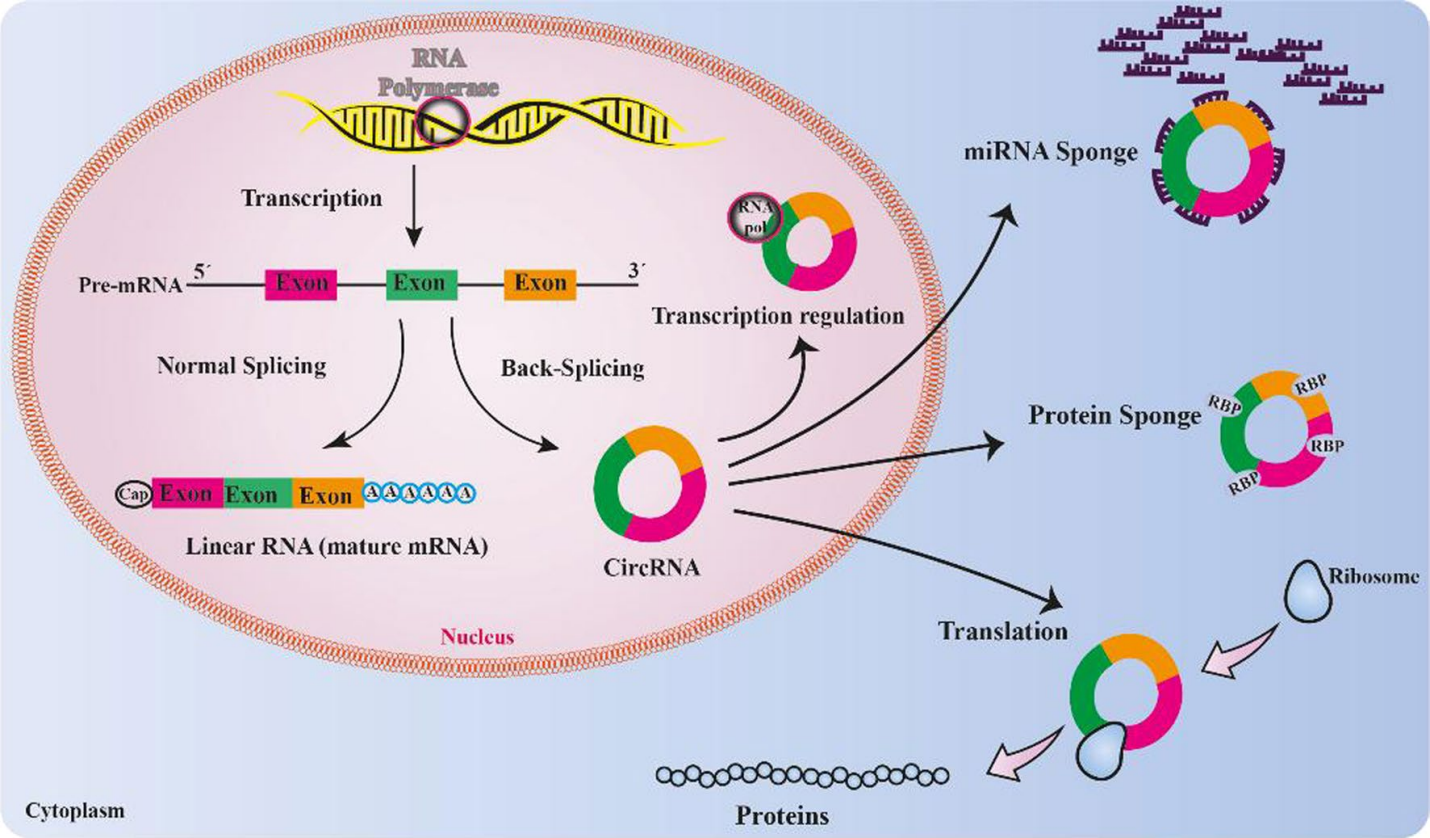
The function of CircRNAs' and their impact on the progression of glioblastoma multiforme

Due to the absence of a 5' cap structure or a 3' poly (A) tail, circRNAs were initially classified as non-coding RNAs that could not be translated into proteins. However, some circRNAs, such as the circ-SHPRH, circ-ZNF609, and circ-Mbl have surprisingly been shown to possess translational capability [51]. The elements required for initiation of the process of translation, including the internal ribosome entry site (IRES), N6-methyladenosine (m6A), and open reading frames (ORFs), are considered as critical elements for the translation of circRNAs [62, 63]. IRES elements drive protein translation by recruiting the 40S subunits of ribosomes in a cap-independent manner [64]. The circ-ZNF609 has an ORF in its structure and can be translated into a protein when is driven by an IRES, in a splicing dependent/cap-independent manner [65]. Furthermore, the circ-SHPRH has an IRES-driven ORF for translating into a functional protein and is expressed in normal human brains to suppresses the glioma tumorigenesis [66]. Similarly, circMbl can also encode a protein in a cap-independent manner [67]. In the absence of IRES, m6A modification facilitates the process of protein synthesis for some circRNAs. Yang et al. reported that consensus m6A motifs were large in number within the circRNAs and a single m6A site, recognized by the reader protein YTHDF3, could deal with the initiation factors eIF4G2 and eIF3A to launch the protein translation processes [62] (Fig. 1).

### CircRNAs in human diseases

CircRNAs may be involved in various human diseases, such as different cancers, cardiovascular diseases (CVDs), neurological disorders, etc. First, circRNAs are greatly expressed in nervous tissues [68], suggesting that they may be contributed to many CNS-related processes, like the synaptic transmission [69], aging [70] neurogenesis, and brain development [71, 72]. Therefore, several researchers have demonstrated that circRNAs play crucial roles in the pathogenesis of a wide variety of neurological defects, such as the Alzheimer's disease (AD) [73], Parkinson’s disease (PD) [74], ischemic stroke [75], cerebral ischemia–reperfusion injury [76], neuropathic pain [77], multiple system atrophy [78], neuro-inflammation [79], major depressive disorder [80], and multiple sclerosis (MS) [81].

Second, circRNAs are also highly expressed in cardiac tissues [82] and cancer [83], migration [84], apoptosis [85], cardiac remodeling [86], endothelial-to-mesenchymal transition (EMT) [87], and atrial fibrillation [88]. Hence, these RNA molecules can also play significant roles in various heart-related diseases, such as the cardiac hypertrophy [89], myocardial infarction (MI) [90], cardiac senescence [91], atherosclerosis [92], and myocardial fibrosis [93].

Consistent with their known functions in the modulation of cell cycles, cell proliferation, and cellular senescence, circRNAs have been implicated in the pathogenesis of cancers [94]. A research team compared the abundance of circRNAs between normal and cancerous tissues, in osteosarcoma, renal cell carcinoma, colorectal adenocarcinoma, lung adenocarcinoma, gastric adenocarcinoma, and hepatocellular carcinoma. The obtained results indicated that the total circRNA levels are negatively correlated with cancer cell proliferation and the down-regulation of circRNAs may be a common phenomenon in cancer progression. However, few circRNAs are significantly expressed in malignancies, which can be the result of high expression levels of the parental genes [95]. Therefore, these circRNAs may act as oncogenes or tumor suppressors in different cancers and affect the cancer phenotypes by diverse mechanisms [96].

#### The expression of circRNAs in GBM

CircRNAs are dynamically expressed in nervous tissues, suggesting their neurospecificity [97]. This phenomenon can be explained by two main reasons; the first refers to the neurospecificity of circRNA-producing genes. Most of the host genes, such as the RTN4, NTRK2, and HOMER1 involved in the production of circRNAs, are specific to brain tissue [98]. The second reason refers to the static status of brain tissue and the circRNAs stability [99]. Thus, the aberrant circRNA expression is evaluated through the progression of various nervous system-related disorders, such as the GBM. By Illumina Hiseq, a powerful sequencing system, Zhu et al. analyzed circRNAs with differential expression levels between the brain tissues of five GBM subjects and five normal individuals. They showed that a total of 1411 differentially expressed circRNAs were detected in tumor tissues from GBM patients. Among these differentially expressed circRNAs, 206 circRNAs were up-regulated and 1205 circRNAs became down-regulated. Besides, the Gene Ontology (GO) analysis and the Encyclopedia of Genes and Genomes (KEGG) pathway analysis indicated that dysregulated circRNAs had played a part in numerous biological processes and signaling pathways in association with cancer development [100]. Yuan et al. diagnosed the GBM-related circRNAs’ expression profiles and matched them with normal ones. The results presented that 2038 circRNAs were differentially expressed in GBM patients, including the 36 up-regulated and 2002 down-regulated genes [101]. Using the circRNA microarray analysis, for three paired IDH-wild type GBM and non-GBM brain tissue samples, Wang et al. confirmed that 615 circRNAs were altered between two groups, of which 254 were up-modulated, while 361 were down-modulated [102]. Afterwards, the validation study indicated that hsa_circRNA_000407, has_circRNA_006169, and has_circRNA_101213 levels were enhanced in IDH-wild type GBM tissues, while the hsa_circRNA_011883, hsa_circRNA_406155, and hsa_circRNA_101341 were silenced [102].

## The role of CircRNAs in GBM: cellular and molecular mechanisms

### CircRNAs and PI3K/Akt/mTOR signaling pathway

The phosphatidylinositol-3'-kinase/protein kinase B/mammalian target of rapamycin (PI3K/Akt/mTOR) pathway is a vital intracellular signaling, which regulates different biological processes such as the cell proliferation, differentiation, apoptosis, angiogenesis, and cell survival [103]. The activation of receptor tyrosine kinases (RTKs), including the EGFR, platelet derived growth factor receptor (PDGFR), and the insulin and insulin-like growth factor 1 (IGF-1) receptor stimulates the corresponding pathway [104]. On the other hand, the PTEN can down-regulate the pathway by converting the PIP3 to PIP2 [105]. Reportedly, the disruption of PI3K/Akt/mTOR signaling is a crucial event in both GBM tumorigenesis and expansion [106]. The improper activation of this pathway in GBM may have several sources, including EGFR mutations (~ 45%), loss of PTEN expression (~ 36%), and PI3K overexpression (~ 15%). In total, these alterations can activate the mentioned pathway in about 88% of GBM individuals [107].

Previous investigations have indicated that circRNAs affect the cell proliferation, differentiation, and migration in several malignancies, such as the liver cancer, gastric cancer, renal cell carcinoma, as well as GBM, through the PI3K/Akt/mTOR signaling. There are some valuable findings in this regard; PENG et al. reported an up-regulated hsa_circ_0010882 expression in plasma specimens of gastric cancer patients, as well as in gastric cell lines. They found that this overexpression promoted the proliferation, migration, and invasiveness of gastric cancer cell lines via activating the PI3K/Akt/mTOR flux [108]. Another valuable finding in this field was concluded by Chen et al., who revealed that the has_circ_0072309 was weakly expressed in renal cancer tissues. They also confirmed that high has_circ_0072309 levels could suppress the proliferation, migration, and invasion, and induced the apoptosis by sponging miR-100 through the PI3K/Akt/mTOR deactivation in renal carcinoma cell lines [109]. Similar outcomes were noted in Lin et al. study, where the aberrant expression of circCDK13 was evaluated in association with the liver cancer tumorigenesis. CircCDK13 overexpression inhibited proliferation, cell cycle progression, migration, and invasion of liver cancer cells, possibly mediated by the JAK/STAT and PI3K/Akt pathways [110].

In the case of miRNA sponges, circTTN acts as a miR-432 sponge to induce the cell proliferation and differentiation of bovine myoblasts through the IGF2/PI3K/Akt signaling. Indeed, miR-432 can inhibit the expression of IGF2 and a group of genes such as the IRS1, PI3K, and phosphoinositide-dependent Kinase-1 (PDK1), all of which are involved in the modulation of PI3K/Akt pathway. Still, the overexpression of xcircTTN may abolish these effects by sponging miR-432 [111]. IGF2 also promotes the cell migration, EMT, and invasion in GBM by initiating the IGF2/PI3K/Akt flux [112]. Previous assessments have reported that IGF2 binding protein 3 (IGF2BP3) is an activator of IGF2/PI3K/Akt signaling pathway [113, 114]. The up-regulated circHIPK3 was also observed in glioma tissues, which had the ability to promote the IGF2BP3 expression by sponging miR-654. According to these findings, circ-HIPK3 overexpression promotes in vivo GBM cell proliferation, invasion, and tumor propagation through the IGF2BP3-induced IGF2/PI3K/Akt signaling pathway [115].

Hsa_circ_0067934, generated from the chromosomal region 3q26, has been shown to become overexpressed in GBM tissues and is contributed to the GBM progression by stimulating of both proliferative and metastatic processes through the activation of PI3K/Akt signaling. The current finding suggests that hsa_circ_0067934, as an oncogene circRNA, may be considered as a novel prognostic biomarker to detect GBM patients as soon as possible [116].

Using high-throughput RNA sequencing, researchers have reported that circ-AKT3 has insignificant expression levels in GBM tissues compared to non-GBM brain tissues. The circ-AKT3 encodes a new protein, containing 174 amino acid residues, called the AKT3-174aa, which blocks the AKT thr-308 phosphorylation and sequential activation via interacting with the active PDK1. Therefore, the circAKT3, as a tumor suppressor, has a negative modulatory role in operating the PI3K/Akt pathway and hampers the GBM cells proliferation, radiation resistance, and tumorigenicity [117].

One of the other GBM-related circRNAs is the circNT5E. MiR-422a, as a tumor suppressive molecule, exerts its suppressive effects on GBM through regulating the PI3K/Akt/mTOR signaling [118]. Wang et al. showed that circNT5E induced the GBM cell proliferation, migration, invasion, and subsequent inhibition of apoptotic flux by sponging the miR-422a [119]. Additionally, He et al. declared that the overexpressed circ-SHKBP1 could up-regulate the forkhead box P1/P2 (FOXP1/FOXP2) by sponging the miR-544a/miR-379 and the further activation of PI3K/Akt pathway in U8 glioma-exposed endothelial cells (GECs). The activated pathway might induce the angiogenic processes in GBM [120].

### CircRNAs and Wnt/β-catenin signaling

Wnt/β-catenin signaling is a highly conserved signaling cascade that controls the fetal growth and adult homeostasis [121]. The Wnt family encompasses cysteine-rich glycoproteins, secreted by the cells located inside the extracellular matrix (ECM) and bind to the cell surface receptors, triggering a variety of intracellular signaling processes, including the canonical and non-canonical pathways [121, 122]. The non-canonical signaling or β-catenin-independent pathway is triggered by the attachment of Wnt ligand to the receptors like the Frizzled (FZD), the RTKs, and receptor tyrosine kinase-like orphan receptor (ROR) in order to regulate the cell polarity and intracellular calcium content [123]. Whereas, the Wnt proteins activate the canonical signaling or beta-catenin-dependent pathway by binding to FZD receptors and low-density lipoprotein receptor-related protein (LRP) [124]. The activation of these receptors makes β-catenin to become accumulated within the cytoplasm and promotes its translocation into the nucleus, where it forms a complex with T-cell factor/lymphoid enhancer factor (TCF/LEF) and induces the transcription of its target genes.

Neural stem cells (NSCs) are the major components of CNS. It has been reported that Wnt signaling is required for the differentiation of NSCs during the CNS development [125]. Thereby, the Wnt signaling hyperactivity can be involved in the pathogenesis of GBM at different levels, including the tumor onset, stem cell maintenance, invasion, and therapeutic resistance. As an example, PLAG2 was demonstrated to up-modulate the expression of WNT6, FZD9, and FZD2, leading to the GBM progression and invasion via the maintenance of stemness properties [126]. The accumulation of β-catenin has also been shown to be positively correlated with the overexpression of programmed cell death ligand 1 (PD-L1) on the surface of tumor cells to induce the GBM immune escape [127].

Aldehyde dehydrogenase isoform 3A1 (ALDH3A1) is a target gene for the canonical Wnt signaling in GBM that plays a significant role in tumor drug resistance [128]. Suwala et al. have shown that in vitro inhibition of Wnt/β-catenin signaling can down-regulate the ALDH3A1 expression to reduce the TMZ resistance in GBM [128]. Over the past few years, several studies have investigated the role of signal molecule-related circRNAs in GBM, which have been reported to exert their modulatory effects on glioma by regulating the Wnt/β-catenin signaling flux. Feng et al. identified the tumor-suppressive functions of circ-ITCH in glioma cells, and revealed that this RNA molecule was negatively regulated in glioma tissues and cell lines. The overexpression of circ-ITCH is achieved at both mRNA and protein levels by sponging the miR-214. This circRNA stimulates the ubiquitination and degradation of phosphorylated disheveled, resulting in the inhibition of Wnt/β-catenin flux to prevent the GBM development. Circ-ITCH exerts the mentioned effects by suppressing the GBM cell proliferation and invasive and migratory capabilities of these cells. Further studies have also indicated the inducing effects of circ-ITCH on the number of apoptotic cells [129, 130].

In this field, Chen and Duan found that hsa_circ_0000177 targeted the mir-638 to increase the expression of FZD class receptor 7 (FZD7), and activated the Wnt signaling in order to induction of malignant glioma behaviors through stimulating the cell proliferation and invasion [131]. Circ_0043278 is another Wnt/β-catenin signaling-related circRNA, which is highly expressed in GBM cell lines and tissues, and is able to directly sponge miR-638 to enhance the Homeobox A9 (HOXA9) expression. Wu et al. declared that HOXA9 overexpression could activate the downstream Wnt/β-catenin pathway and enhanced the GBM evolution through the in vitro inhibition of cell proliferation, migration, and invasion, and in vivo suppression of tumorigenesis [132].

CZNF292 is a circRNA that has been shown to be expressed in hypoxic conditions and modulates the GBM tube formation by activating the Wnt/β-catenin pathway [133, 134]. Yang et al. reported that cZNF292 knockdown could suppress the proliferation, angiogenesis, and cell cycle progression in glioma cells by modulating the Wnt/β-catenin pathway. The cZNF292 quelling also decreased the activity of a group of transcription factors, such as the AP1, SP1, E2F1, HIF1, NF-Bb, STAT3, and STAT5, all of which play key roles in tumor tube formation [134]. Sp1, as a substantial stemness-related transcriptional factor and stress-responsive factor, protects the GBM cells against TMZ- and stress-induced death [135]. SP1 can enhance the circ-000 × expression through binding to the circ-0001730 host gene EPHB4 promoter. Moreover, circ-0001730 activates the Wnt/β-catenin flux through the miR-326/Wnt7B axis, and enhances the migration and proliferation of GBM cells [136]. Previous evaluations have shown that sirtuin1 (SIRT1) can also exert some stimulatory effects on the Wnt/β-catenin pathway [137, 138]. On the other hand, miR-326 is able to bind to SIRT1 in order to reduce its expression. Interestingly, the circ_0082374 can up-regulate the SIRT1 expression in a miR-326-dependent manner, and turns on the Wnt/β-catenin signaling in A172 and U251 glioma cell lines [139].

### CircRNAs and MAPK signaling

Mitogen-activated protein kinases (MAPKs) are highly conserved serine/threonine protein kinases that regulate various cancer cell-related processes such as proliferation, differentiation, transformation, stress response, apoptosis, cell survival, and death [140]. There are three major subgroups of MAPKs, including the c-Jun N-terminal kinase (JNK), extracellular signal-regulated kinase (ERK), and p38 [141]. The ERK signaling is mainly activated by growth factors such as the epidermal growth factor (EGF), while the JNK and p38 pathways are switched on in response to oxidative stress stimuli and inflammatory cytokines [142]. Each MAPK cascade consists of at least three members; a MAPK kinase kinase (MAP3K), a MAPK kinase (MAP2K), and a MAP kinase (MAPK). MAP2Ks and MAPKs become phosphorylated, and subsequently activated by MAP3K and MAP2K, respectively [140]. Triggered MAPKs also phosphorylate different downstream proteins, including transcription factors such as the p53, SMAD3, c-FOS, c-MYC, c-JUN, ATF1, ATF2, USF1, HIF-1a, and HSF1, which thereby regulate the expression of different target genes [143]. Recent evaluations have proposed that MAPK mutations are involved in various cancers, such as the GBM. Many of these cancer-related mutations have been found in Ras, as a key player in GBM pathogenesis [144–146]. Consistent with this valuable finding, it has also been reported that circRNAs can affect GBM-associated cellular processes by modulation of MAPKs signaling.

One of these circRNAs is circ-TTBK2, which Zheng and his collaborators have indicated its overexpression in both GBM tissues and cell lines. Surprisingly, if circ-TTBK2 is knocked down, miR-217 up-modulation can significantly inhibit the processes like cell proliferation, migration, and invasion, and accelerates the apoptotic events. Further investigations also presented that circ-TTBK2 might have the ability to induce the expression of hepatocyte nuclear factor 1 (HNF1), as a crucial carcinogen in glioma cells, through sponging the miR-217 and further promotion of the GBM expansion. Scanning the promoter’s sequence has exhibited that HNF1β has a binding site on Derlin-1 promoter [147]. Previous analyses have appeared that Derlin-1 exerts its oncogenic effects by activating the ERK pathway [148, 149]. Therefore, the circ-TTBK2/miR-217/HNF1/Derlin-1 loop is considered to be impressively involved in malignant growth of GBM cells through the activation of ERK signaling [147].

Circ-MAPK4, also known as has_circ_0047688, is a special member of circRNA family, which is positively correlated with the pathological stage of GBM. He et al. declared that circ-MAPK4 promoted GBM cell survival and inhibited the apoptosis through suppressing the phosphorylation of p38/MAPK, as an apoptosis inducer and sequential inhibitor, working through miR-125a-3p sponging [150]. Wei et al. worked on another circRNA in this field. They stated that circASAP1 could be overexpressed in recurrent GBM tissues and significantly correlated with TMZ-resistant phenotype. CircASAP1 is actually a miRNA sponge and exerts its carcinogenic impacts by targeting the miR-502-5p, resulting in NRAS dysmodulation and MEK1/ERK1/2 signaling activation. The outcomes would be the promotion of cell growth and inhibition of apoptosis, and final TMZ resistance [151]. Thus, it can be concluded that circASAP1 may involve in GBM chemoresistant processes.

Through a mechanistic evaluation, researchers found the cytoplasmic localization and molecular sponge role of circ-PITX1. They have revealed that this circRNA interacts with miR-379-5p, as well as the MAP3K2, in order to block the cell proliferation and accelerate the apoptotic flux, leading to the GBM development [152]. Based on a group of relevant investigations, miR-7-5p has some remarkable tumor-suppressive impacts on GBM by modulating the EGFR, Raf/MEK/ERK, and PI3K/AKT signaling pathways [153, 154]. Interestingly, Li et al. noted that miR‐7‐5p was a direct target of circ-U2AF1 (hsa_circ_0061868) and neuro‐oncological ventral antigen 2 (NOVA2) is a direct target of miR‐7‐5p. It has been reported that circ-U2AF1 was positively associated with the malignant progression of GBM and the elevated cell proliferation, migration, and invasion, which were done by miR‐7‐5p down-modulation and NOVA2 up-regulation [155].

### CircRNAs and angiogenic flux

Angiogenesis is a physiological process through which new capillaries are generated from previous arteries. Physiological angiogenesis is a highly regulated process and is critical for growth, as well as wound healing and tissue formation [156]. The induction of angiogenesis depends on the balance between angiogenic and anti-angiogenic factors. Therefore, the overexpression of angiogenic factors causes unregulated angiogenesis and makes the tumors to transform from a benign to a malignant state, leading to the tumor growth and expansion [157]. In GBM, the overexpression of factors such as the vascular endothelial growth factor (VEGF), fibroblast growth factors (FGFs), HIF-1, transforming growth factor-β (TGF-β), and angiopoietins (Angs) induces the angiogenic processes [158]. These factors are up-regulated by the activation of oncogenes, loss of tumor suppressor gene expression, and/or during the hypoxic conditions [159]. In this regard, circRNAs have been shown to affect a number of angiogenic processes to modulate the GBM tumorigenesis.

Neuropilin-1 (NRP-1) is a multifunctional receptor that is expressed in different human cancerous tissues, including the GBM, and its expression level is associated with tumor growth [160]. Recent evaluations have shown that NRP-1 induces the angiogenesis through the NRP-1/VEGF/VEGFR2 complex formation [161, 162]. In addition to VEGF and its receptors, NRP-1 also binds to other proteins such as the PDGF, TGF-β1, and GIPC1, which their expression levels are positively associated with both angiogenesis and tumorigenesis in glioma cells [163–165]. Zhang et al. identified miR-124-3p, which was differently expressed in GBM; they found that miR-124-3p could specifically bind to the 3' UTR region of NRP-1 and suppressed its expression. Thus, the overexpression of miR-124-3p, as an upstream suppressor of NRP1, inhibited the GBM cell angiogenesis, leading to the suppression of proliferation and migration. On the other hand, they led to induction of apoptosis and cell cycle arrest in GBM [166], while circ-HIPK 3 could invert these effects by sponging miR-124-3p [167].

RBPs are reported to be involved in the operation of tumor angiogenesis by interacting with circRNAs. As an example, serine and arginine rich splicing factor 10 (SRSF10), belonging to the SR protein family, binds to the 5′-end and 3′-end of circ-ATXN1 pre-mRNA and increases the circ-ATXN1 biogenesis and inhibits the SRSF10, resulting in a dramatically repression of the tube formation in glioma endothelial cells (GECs). Moreover, circ-ATXN1 attaches to the miR-526b-3p and blocks the negative regulatory effect of miR-526b-3p on MMP2 and VEGFA, subsequently increases the GBM angiogenesis [168]. Another study showed that MOV10 increased the circ-DICER1 biogenesis through binding to circ-DICER1. Circ-DICER1 targets the miR-103a-3p/miR-382-5p and promotes the ZIC4 expression. ZIC4 by itself, can up-regulate the downstream Hsp90β, which increases the tube formation of GECs by activating PI3K/Akt pathway. Hence, MOV10 modulates the GBM angiogenesis trough targeting the circ-DICER1/miR-103a-3p (miR-382-5p)/ZIC4/Hsp90β pathway [169]. Jiang et al. described the up-modulation of circRNA ARF1 (cARF1) in GECs. It positively regulates the expression of ISL2 by sponging the miR-342–3p. ISL2 stimulates the expression of VEGFA at transcriptional level in glioma stem cells (GSCs), inducing the angiogenesis via ERK signaling. ISL2 also induces the U2AF2 expression, leading to an increase in cARF1 expression in GSCs. Thereby, the U2AF2/cARF1/miR-342–3p/ISL2 feedback loop modulates the angiogenic flux and accelerates the GBM proliferation and invasion [170].

Similar outcomes were observed for the FUS/circ_002136/miR-138-5p/SOX13 feedback loop, which played a vital role in regulating the GBM angiogenesis [171]. CircSMARCA5 has particular protein binding-sites for the SRSF1 splicing factor [172]. SRSF1 increases the expression of VEGFA angiogenic isoforms by binding to the proximal splicing site (PSS) of VEGFA pre-mRNA. CircSMARCA5 binds to the SRSF1 and decreases its expression, subsequently suppresses the angiogenesis [173]. According to the study performed by Barbagallo et al., circSMARCA5 is down-regulated, while the SRSF1 and VEGFA are up-regulated in GBM cells, leading to the induction of GBM angiogenesis and progression [173].

### CircRNAs and metastatic pathways

Cancer metastasis is the process by which cancer cells separate from the primary tumor, settle and grow in a different or secondary site [174]. In GBM, intracranial metastasis occurs frequently in which tumor cells migrate along the blood vessels, meninges, and nerve tracts, while extracranial metastasis is extremely rare, affecting mainly pleura and the lungs [175, 176]. The rarity of this phenomenon can be due to under diagnosis and short survival of the patients [177]. The intracranial metastasis consists of integrated biochemical processes, including the detachment of the tumor cells from the original site, attachment to the ECM, degradation of the ECM, and migration [178]. The overexpression of IL-6, IL-8, CD44, and MMP-2/-9 induces the intracranial metastasis in GBM [179]. Cumulative evidence has also shown that circRNAs are associated with cancer metastasis [180, 181] and an increase in the number of functional circRNAs have been identified in GBM metastasis.

In this regard, circ-EPB41L5 (YMO1) is a direct target for miR-19a and is a critical tumor suppressive molecule that interacts with RhoC and inhibits its expression. Furthermore, circ-EPB41L5, which is down-regulated in GBM, is a circRNA gene that can inactivate the miR-19a, thereby inhibits the phosphorylation of the Akt through the EPB41L5 overexpression, and subsequently can repress the invasion and metastasis of glioma cells to inhibit the GBM tumorigenesis. Hence, these findings indicate that the circ-EPB41L5/miR-19a/EPB41L5/p-Akt regulatory axis plays a prominent role in GBM expansion [182]. Zhou *et al*. observed that hsa_circ_0008344 was greatly expressed in IDH1 wild-type GBM and its knockdown could also cease the GBM cell migration, invasion, and proliferation, and promoted the cell apoptosis [21]. Hsa_circ_01844 was also reported to be significantly decreased in GBM tissues and its overexpression significantly stimulated the apoptosis and suppressed the migration, proliferation, and colony formation in GBM cells [183].

The major process responsible for regulation of metastasis is called EMT, where the cells lose their cell polarity and cell-to-cell or cell-to-matrix adhesion, become mesenchymal stem cells with migratory and invasive capabilities [184]. MiRNAs play a fundamental role in EMT modulation, and circRNAs, as miRNA sponges, may be involved in this regulation through the endogenous competitive mechanisms to influence the EMT-related parameters. High mobility group box 3 (HMGB3) has been shown to induce the growth and migration of malignant cells by operating the MAPK pathway [185]. A study performed by Chen *et al*., has presented that HMGB3 is a direct target for miR-628-5p, and circ-0001801 acts as a negative regulator of mir-628-5p. This study also showed the up-regulation of circ-0001801 and HMGB3, as well as the down-regulation of miR-628-5p in GBM. When the circ-0001801 is eliminated, miR-628-5p overexpression reduces the GBM cell proliferation, migration, invasion, and EMT [186].

**Fig. 2:**
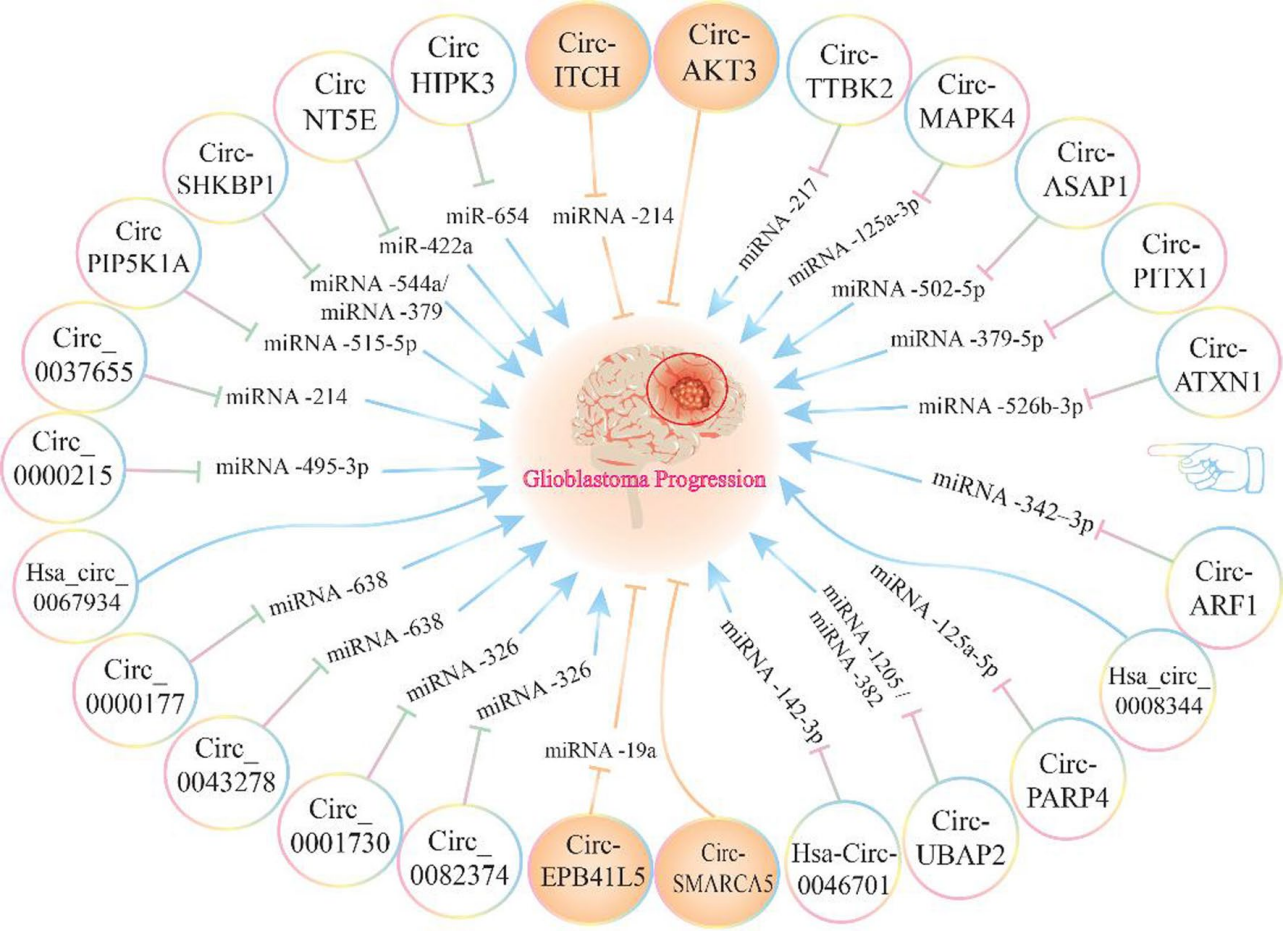
The importance of CircRNAs' and their impact on the progression of glioblastoma multiforme

Another overexpressed circRNA in GBM tissues is circPVT1. If circPVT1 is silenced, it can significantly reduce the viability and migration capability and induces the apoptotic processes via up-regulating the miR-199a-5p. Further investigation have also indicated that EGF-induced EMT was repressed after circPVT1 silencing, suggesting that circPVT1 promotes the GBM metastasis in an EMT-induced manner [187]. Through another analysis, Zhou *et al*. confirmed the elevated expression of circPARP4 in GBM and showed that circPARP4 obviously promoted the glioma cell proliferation, migration, invasion, and EMT. This study also indicated that circPARP4 exerted its oncogenic effects by sponging the miR-125a-5p and through the regulation of FUT4 [188]. We know MMPs as zinc-dependent endopeptidases, produced by various cells, including the tumor cells, and induce both EMT and metastasis by cleaving the cell surface proteins and ECM components [189]. Wang et al. identified that circ-MMP9 was switched on in GBM. Additionally, the circ-MMP9 overexpression triggers the migration and invasion abilities of GBM cells by targeting the miR-124 [56]. Circ-MMP9 has also been shown to bind to ARE/poly (U)-binding/degradation factor 1 (AUF1) and mir-149 to protect MMP9 mRNA from degradation, increasing the MMP-9 expression and facilitates the invasive and metastatic processes [190] (Fig. 2 and Table 1).

**Table 1:** The effects of circRNAs on molecular and signaling mechanisms involved in GBM progression

CircRNA	Function	Signaling pathway	Expression	Target GBM-related cellular process	References
circHIPK3	Sponging miRNA -654	IGF2/PI3K/Akt	Upregulated	Induces the proliferation, invasion, and tumor propagation	[115]
Hsa_circ_0067934	–	PI3K/AKT	Upregulated	Induces cancer cell proliferation and metastasis	[116]
circ-AKT3	–	PI3K/AKT	Downregulated	Inhibits the proliferation, radiation resistance and tumorigenicity	[117]
circNT5E	Sponging miRNA -422a	SOX4/PI3KCA	Upregulated	Promoted proliferation, migration, and invasion/Inhibits the apoptosis	[119]
cir-SHKBP1	Sponging miRNA -544a/miRNA -379	PI3K/AKT	Upregulated	Induces angiogenesis	[120]
CircPIP5K1A	Sponging miRNA -515-5p	TCF12 and PI3K/AKT	Upregulated	Induces proliferation, invasion, and EMT/Inhibited apoptosis	[205]
circ_0037655	Sponging miRNA -214	PI3K	Upregulation	Induces the viability and invasion of glioma cells	[206]
Circ_0000215	Sponging miRNA -495-3p	CXCR2/PI3K/Akt	Upregulated	Induces the proliferation, invasion and EMT/Inhibited apoptosis of glioma cells	[207]
circ-ITCH	Sponging miRNA -214	Wnt	Downregulated	Inhibited proliferation, invasion, and migration/Promoted the apoptosis	[129]
circ_0000177	Sponging miRNA -638	Wnt	Upregulated	Induces Proliferation, invasion, glioma growth malignant glioma behaviors	[131]
Circ_0043278	Sponged miRNA -638	Wnt	Upregulated	Promoted the cell migration, proliferation, and invasion	[132]
Circ 0,001,730	Sponged miRNA -326	Wnt/β-catenin	Upregulated	Induces the migration and proliferation of glioma cells	[136]
circ0082374	Sponging miRNA -326	Wnt/β-catenin	Upregulated	Induces viability, migration, invasion and glycolysis in glioma cells	[139]
circ-TTBK2	Sponging miRNA -217	MAPKs	Upregulated	Induces the cell proliferation, migration, and invasion/Inhibited apoptosis	[147]
circ-MAPK4	Sponging miRNA -125a-3p	MAPKs	Upregulated	Promoted survival and inhibited apoptosis	[150]
circASAP1	Sponging miRNA -502-5p	MAPKs	Upregulated	Promotes cell growth and inhibition of apoptosis	[151]
Circ-PITX1	Sponging miRNA -379-5p	MAPKs	Upregulated	Promoting the proliferation/Inhibit apoptosis	[152]
circ-ATXN1	Sponging miRNA -526b-3p	–	Upregulated	Increased glioma angiogenesis	[168]
circ ARF1	Sponging miRNA -342–3p	ERK	Upregulated	Induced angiogenesis	[170]
CircSMARCA5	–	–	Downregulated	Suppresses angiogenesis	[173]
circ-EPB41L5	Sponging miRNA -19a	circ-EPB41L5/miR-19a/EPB41L5/p-AKT	Downregulated	Inhibition the proliferation, invasion, migration, metastasis and tumorigenesis	[182]
hsa_circ_0008344	–	–	Upregulated	Induced cell migration, invasion and proliferation, and inhibition the apoptosis	[21]
circPARP4	Sponging miRNA -125a-5p	–	Upregulated	Promoted proliferation, migration, invasion, and EMT	[188]
Circ-UBAP2	Sponging miRNA -1205 and miRNA -382	–	Upregulated	Promoted cell proliferation, migration, invasion, and reduced apoptosis	[208]
hsa_circ_0046701	Sponging miRNA -142-3p	–	Upregulated	Promoted cell proliferation and invasion	[209]

CircRNA, circular RNA; miRNA, microRNA

## The application of circRNAs in GBM

### CircRNAs as diagnostic and prognostic biomarkers

It is well established that most cancers would be treated if diagnosed timely [191–193]. Common cancer diagnostic methods such as computed tomography (CT) scan, magnetic resonance imaging (MRI), and histopathology are invasive and/or expensive. On the other hand, the molecular pathogenesis of GBM is very complex due to the cell heterogeneity and multiple genetic alterations. Hence, looking for diagnostic and prognostic biomarkers in body fluids and cancerous tissue can be a helpful proceeding. Researches have declared that circRNAs are widely present in a variety of biological specimens, including blood samples, cerebrospinal fluid (CSF), urine, saliva, breast milk, various tissues, and also exosomes [51, 194]. In addition to their extensive distribution, the unique structure of circRNAs protects them from degradation and increases their stability and half-life [195]. Thus, these RNA molecules seem to be great diagnostic markers through the early stages of GBM.

In this context, it has been shown that hsa_circ_0043278, circ-PITX1, and circ_0074027 are significantly up-modulated in GMB cell lines and tissues, converting them into desirable biomarkers in both GBM diagnosis and prognosis [22, 132, 152]. Wang et al. reported that the suppressed circ_0001649 was positively associated with tumor size and WHO grade, suggesting that circ_0001649 might be an independent prognostic marker following the surgical interventions. Furthermore, the circ_0001649 overexpression promotes the apoptosis by targeting the Bcl-2/caspase-3 pathway [196].

Zhu and collaborators have screened the expression profiles of circRNAs in specimens obtained from GBM patients, using the Illumina Hiseq. Through this valuable investigation, 1411 circRNAs were found to be differentially expressed of which 1205 circRNAs were down-modulated and 206 were up-regulated. They found that circ-BRAF was remarkably decreased in patients with high pathological grades (WHO III & IV) than those with lower grades (WHO I & II). These findings indicate that circ-BRAF, as a biomarker, may help the prediction of pathological grade and prognosis in GBM patients [100]. Circ-0001445 (circ-SMARCA5), which is significantly down-modulated in GBM biopsies, is negatively correlated with glioma’s histological grade, helps the GBM grading. This suggests the circ-SMARCA5 as another diagnostic biomarker [172].

Recent assessments have identified peptides/proteins encoded by circ-RNAs as potential biomarkers in diagnosis of GBM. SHPRH-146aa, encoded by circ-SHPRH, is greatly found in normal human brain, while becomes decreased in GBM. This novel protein protects the full-length SHPRH from DTL-induced ubiquitination, leading to an increase in full-length SHPRH’s half-life and induces the protein’s tumor suppressive functions. SMO-193aa, encoded by circ-SMO, as another protein in this categorization, is essential for hedgehog signaling activation and promotes the self-renewal processes, cell proliferation, and tumorigenicity. The SHPRH-146aa overexpression and SMO-193aa silencing are negatively associated with patients’ short survival period, indicating the clinical application of these proteins [197, 198].

### CircRNAs as therapeutic targets

A number of circRNAs have been shown to affect critical biological processes associated with GBM, including cell proliferation, cell apoptosis, migration, invasion, and metastasis. Accordingly, therapeutic strategies targeting circRNAs are expected to provide a new perspective to cure the GBM. A recent evaluation, performed by Li et al. has indicated that circ_0001946, a miR‐671‐5p sponge, was suppressed in GBM cells, promoting the cerebellar degeneration related protein 1 (CDR1) gene expression. This study also showed that CDR1 reduced the proliferation, migration, and invasion, and increased the apoptosis in GBM cells. Thereby, circ_0001946 seems to have anti-cancer functions, converting it into a promising target for GBM treatment [199]. As a tumor suppressive circRNA, CDR1 binds to p53 in DNA-binding region (DBD) and their interaction disrupts the p53 to MDM2 attachment, preventing the formation of the p53/MDM2 complex and also protects the p53 against ubiquitination and subsequent degradation. Thus, in GBM, the down-regulated CDR1 may increase the tumorigenesis due to the p53 inactivation [200].

Some circRNAs are up-regulated in GBM, and then promote the oncogenic processes. For example, circFOXO3, which is highly expressed in GBM tissues, accelerates the proliferation and invasion of GBM cells by targeting the miR-138-5p and miR-432-5p, in order to abnormally express the nuclear factor of activated T-cells 5 (NFAT5) [201]. It has been revealed that circular E-cadherin (circ-E-Cad), which encodes the oncogenic variant of E-cadherin, is significantly overexpressed in GBM and activates the EGFR (in the absence of EGF) through the EGFR–STAT3 signaling, resulting in the maintenance of GSCs properties and the promotion of GBM tumorigenicity [202]. If the expression of oncogenic circRNAs is inhibited, then it may be possible to use them as therapeutic targets.

TMZ is commonly used to treat GBM patients, who have undergone the resection surgery. Using TMZ after the surgery prevents from the GBM recurrence and prolongs the patient’s survival. However, the GBM chemoresistance limits the TMZ therapeutic effects [203]. Rao *et al*. found that circ‐0,007,874 (circMTO1) was overexpressed in TMZ‐resistant GBM cells and tissues. Moreover, the circMTO1 overexpression remarkably decreased in vivo and in vitro TMZ‐resistance of these cells by suppressing the cell proliferation and inducing the apoptotic flux. Further analyses also revealed that miR‐630 was targeted by circMTO1 in GBM cells and its knockdown also reduced the TMZ‐resistance. Therefore, circMTO1 can reverse the TMZ-resistant property of GBM cells through the regulation of miR‐630, providing a novel and potential target to manage GBM [204]. Another group of researchers have shown that the silenced circASAP1 can noticeably trigger the TMZ sensitivity via sponging the miR-502-5p, which can block the NRAS expression (151). Nowadays, several researchers are working hard to convert circRNAs into novel and effective therapeutic targets to eradicate the GBM, as one of the most life-threatening human malignancies.

## Conclusions and perspectives

Despite the surgical interventions, followed by chemotherapy and radiotherapy, the survival rate of GBM patients has remained very low and most patients do not unfortunately survive more than two years. The other issue is the GBM’s histological heterogeneity and multiplicity of underlying molecular mechanisms, making it resistant to common radiotherapy and chemotherapeutic approaches. Consequently, targeted therapies towards the intracellular signaling pathways would be potentially effective strategies to defeat GBM. Not long ago, several analyses have examined the circRNAs’ biological functions in different cancers. They presented that these RNA molecules could influence diverse biological processes in association with the GBM expansion, including cell proliferation, apoptosis, invasion, and treatment resistance. Many of these effects are obtained by operating of critical molecular mechanisms and/or signaling pathways. Some of these circRNAs can positively modulate the crucial pathways, such as the PI3K/Akt/mTOR signaling, Wnt/β-catenin pathway, and MAPKs cascade, while some negatively regulates them. The other ones can be involved in cancer development-related processes, including the angiogenesis and metastatic flux. According to aforementioned findings and extensive distribution of circRNAs, as they can be found in various biological specimens, a novel perspective has been proposed for both diagnosis and treatment of GBM. However, in comparison to mRNAs and miRNAs, there is a significant gap in our current understanding of circRNAs, and thus further studies are required to identify the precise mechanisms of GBM-circRNAs relationship. Currently, detecting circRNAs in cancer patients is mainly done by analyzing tissue specimens, which is an invasive method. Thereupon, it is critical to know how to employ non-invasive methods such as analyzing blood, urine, saliva, etc. Additionally, it is worth noting to investigate the association of different circRNAs with common GBM biomarkers. The development of newly found circRNA-identifying methods is improving the GBM circRNA-based diagnostics and therapeutic approaches, which will be a milestone in eradicating of this life-threatening malignancy.

## Data Availability

Not applicable.

## Abbreviations

ALDH3A1: Aldehyde dehydrogenase isoform 3A1
Angs: Angiopoietins
ECM: Extracellular matrix
EGFR: Epidermal growth factor receptor
FAK: Focal adhesion kinase
GBM: Lioblastoma multiforme
HIF-1α: Hypoxia-inducible factor-1α
IDH1/2: Isocitrate dehydrogenase1/2
IGF-1: Insulin-like growth factor 1
IRES: Internal ribosome entry site
MDM2: Mouse double minute 2
NcRNA: Non-coding ribonucleic acids
NF1: Neurofibromin-1
NSCs: Neural stem cells
PDGFR: Platelet derived growth factor receptor
RTKs: Tyrosine kinases
SnRNPs: Small nuclear ribonucleic proteins
TMZ: Temozolomide
